# Climate change and health: Why should India be concerned?

**DOI:** 10.4103/0019-5278.50717

**Published:** 2009-04

**Authors:** J. P. Majra, A. Gur

**Affiliations:** Department of Community Medicine, K. S. Hegde Medical Academy, Mangalore, India; 1 Department of Periodontics, A. B. Shetty Memorial Institute of Dental Sciences, Mangalore, India

**Keywords:** Climate change, health effects, India, public health

## Abstract

Overwhelming evidence shows that climate change presents growing threats to public health security – from extreme weather-related disasters to wider spread of such vector-borne diseases as malaria and dengue. The impacts of climate on human health will not be evenly distributed around the world. The Third Assessment Report (Intergovernmental Panel on Climate Change-2001) concluded that vulnerability to climate change is a function of exposure, sensitivity, and adaptive capacity. Developing country populations, particularly in small island states, arid and high mountain zones, and in densely populated coastal areas are considered to be particularly vulnerable. India is a large developing country, with the Great Himalayas, the world's third largest ice mass in the north, 7500 km long, and densely populated coast line in the south. Nearly 700 million of her over one billion population living in rural areas directly depends on climate-sensitive sectors (agriculture, forests, and fisheries) and natural resources (such as water, biodiversity, mangroves, coastal zones, grasslands) for their subsistence and livelihoods. Heat wave, floods (land and coastal), and draughts occur commonly. Malaria, malnutrition, and diarrhea are major public health problems. Any further increase, as projected in weather-related disasters and related health effects, may cripple the already inadequate public health infrastructure in the country. Hence, there is an urgent need to respond to the situation. Response options to protect health from effects of climate change include mitigation as well as adaptation. Both can complement each other and together can significantly reduce the risks of climate change.

## INTRODUCTION

Climate change is a significant and emerging threat to public health. Hence, it is finding an increasingly central position on the international agenda as most recently evidenced by the Nobel Prize awarded to the former US Vice President, Al Gore, and a team of UN experts under the chairmanship of Dr. Rajendra K. Pachauri (Director General, The Energy and Resources Institute, New Delhi) for their work on the subject. In 2008, the World Health Organization (WHO) focused on the need to protect health from the adverse effects of climate change. The World Health Day – 2008 theme “Protecting health from climate change” raises the profile of health dangers posed by global climate variability and change. It was selected because overwhelming evidence shows that climate change presents growing threats to international public health security.

## SCOPE

This article describes the process of global climate change, its current and future impacts on human health in general and India in particular, and how we can lessen those adverse impacts by mitigation and adaptation strategies.

## GLOBAL CLIMATE CHANGE

Climate change occurs over decades or longer time scales. Until now, changes in the global climate have occurred naturally, across centuries or millennia, because of continental drift, various astronomical cycles, variations in solar energy output, and volcanic activity. Over the past few decades, it has become increasingly apparent that human actions are changing atmospheric composition, thereby causing global climate change.[1] Humankind's activities are altering the world's climate by increasing the atmospheric concentration of energy-trapping gases (greenhouse gases [GHGs]), thereby amplifying the natural “greenhouse effect” that makes the Earth habitable. These GHGs comprise, principally, carbon dioxide (mostly from fossil fuel combustion and forest burning) plus other heat-trapping gases such as methane (from irrigated agriculture, animal husbandry, and oil extraction), nitrous oxide, and various human-made halocarbons. According to the Fourth Assessment Report (2007)[1] of the Intergovernmental Panel on Climate Change (IPCC), the observed effects include:

1. The global average surface temperature has increased by approximately 0.65°C over the last 50 years.
2. Eleven of the last 12 years (1995–2006) rank among the 12 warmest years since records began in the 1850s.
3. The rates of warming and of sea level rise have accelerated in recent decades.
4. Many areas, particularly mid- to high-latitude countries, have experienced increases in precipitation and there has been a general increase in the frequency of extreme rainfall.
5. In some regions, such as parts of Asia and Africa, the frequency and intensity of droughts have increased in recent decades.
6. The frequency of the most intense tropical cyclones has increased in some areas, such as the North Atlantic, since the 1970s.

As we continue to change atmospheric composition, climatologists forecast further warming during the coming century and beyond. The IPCC has made the following projections for the next century:

1. Global mean surface temperature will rise by 1.1–6.4° C, depending partly on future trends in energy use. Warming will be greatest over land areas and at high latitudes.
2. Heat waves, heavy precipitation events, and other extreme events will become more frequent and intense.
3. Sea level rise is expected to continue at an accelerating rate.

## IMPACT OF CLIMATE CHANGE ON HUMAN HEALTH

Our personal health may seem to relate mostly to prudent behavior, heredity, occupation, local environmental exposures, and health-care access, but sustained population health requires the life supporting “services” of the biosphere. Populations of all animal species depend on supplies of food and water, freedom from excess infectious disease, and the physical safety and comfort conferred by climatic stability. The world's climate system is fundamental to this life support. A changing climate is likely to affect all these conditions and hence have a powerful impact on human health and well-being.[2] In its Third Assessment Report, the United Nation's IPCC concluded that “climate change is projected to increase threats to human health.” Climate change can affect human health directly (e.g., impacts of thermal stress, death/injury in floods and storms) and indirectly through changes in the ranges of disease vectors (e.g., mosquitoes), water-borne pathogens, water quality, air quality, and food availability and quality. Global climate change is, therefore, a newer challenge to ongoing efforts to protect human health.[3]

## WHY SHOULD INDIA BE CONCERNED?

The Third Assessment Report (IPCC-2001) concluded that vulnerability to climate change is a function of exposure, sensitivity, and adaptive capacity. India is a large developing country, with the Great Himalayas, the world's third largest ice mass in the north, 7500 km long, and densely populated coast line in the south. Nearly 700 million of her one billion population living in rural areas directly depends on climate-sensitive sectors (agriculture, forests, and fisheries) and natural resources (such as water, biodiversity, mangroves, coastal zones, grasslands) for their subsistence and livelihoods. Further, the adaptive capacity of dry land farmers, forest dwellers, fisher folk, and nomadic shepherds is very low.[4] Climate change is likely to impact all the natural ecosystems as well as socioeconomic systems, as shown by the National Communications Report of India to the United Nations Framework Convention on Climate Change (UNFCCC).[5]

The latest high-resolution climate change scenarios and projections for India, based on the Regional Climate Modeling system, known as PRECIS, developed by the Hadley Center and applied for India using IPCC scenarios A2 and B2,[6] show an annual mean surface temperature rise by the end of the century, ranging from 3 to 5°C under the A2 scenario and 2.5 to 4°C under the B2 scenario, with warming more pronounced in the northern parts of India. A 20% rise in all India summer monsoon rainfall and further rise in rainfall is projected over all states except Punjab, Rajasthan, and Tamil Nadu, which show a slight decrease. Extremes in maximum and minimum temperatures are also expected to increase and similarly extreme precipitation also shows substantial increases, particularly over the west coast of India and west central India. Rapid mountain glacier retreat has been documented in the Himalayas, meltwater from the Himalayan glaciers contributing a sizeable portion of river flows to the Ganges, Brahmaputra, Indus, and other river systems.[7] Public health, to a large extent, depends on safe drinking water, sufficient food, secure shelter, and good social conditions. A changing climate is likely to affect all these conditions.

Some of the current and future health effects include:

### Health effects of extreme temperatures

Extremes of temperature can kill. While Himachal Pradesh and Uttaranchal experienced a cold wave, other parts in the country were subjected to heat wave. In 1998, the heat wave in Orissa was recorded as one of the worst, claiming more than 2000 lives.[8] 1998 was the warmest year globally.[9] Andhra Pradesh reeled under heat wave in 2003, killing 1421 people, which is an all-time high in the history of Andhra Pradesh.[10] Effects of heat wave were also observed in Uttar Pradesh, Haryana, Punjab, Rajasthan, Gujarat, Bihar, and Orissa in 2003. In June 2005, Orissa recorded the highest temperature of 46.3°C in Bhubaneswar of the last 33 years, which is 10° above normal,[11] leading to a heat wave. This is not limited to India only. In July 1995, a heat wave in Chicago, USA, caused 514 heat-related deaths (12 per 100,000 population) and 3300 excess emergency admissions. The record high temperatures in Western Europe in the summer of 2003 were associated with a spike of an estimated 70,000 more deaths than the equivalent periods in previous years. Most of the excess deaths during times of thermal extreme are in persons with pre-existing disease, especially cardiovascular and respiratory disease. The very old, the very young, and the frail are the most susceptible.[12] Extremes in maximum and minimum temperatures are also expected to increase. Therefore, it is anticipated that there will be an increase in the number of deaths due to greater frequency and severity of heat waves.

### Health effects of extreme weather events

Extreme weather events such as severe storms, floods, and drought have claimed thousands of lives during the last few years and have adversely affected the lives of millions and cost significantly in terms of economic losses and damage to property. India and the subcontinent saw five of the 20 major natural calamities recorded worldwide in terms of victims. Orissa is no stranger to cyclones, but the 1999 cyclone was unprecedented for the sheer severity, with wind speed reaching over 300 km/h, leaving nearly 10,000 dead, and has gone down in history as the Super cyclone.[13] In 2003, floods claimed thousands of lives and rendered millions of people homeless in Assam, Bihar, West Bengal, Orissa, Uttar Pradesh, Himachal Pradesh, Rajasthan, and Gujarat. Severe drought conditions is most of the north west, major parts of north India, north east India, and parts of Andhra Pradesh, the Telangana and Rayalseema regions, and parts of Tamil Nadu destroyed crops to the tune of USD 25 million, with many starvation deaths being reported. Floods are an annual feature in Bihar, but the 2004 floods was unique for its severity. Recent climate emergencies in India included a heat wave in Orissa (2004), a cold wave in Uttaranchal and Uttar Pradesh (2004), a tsunami affecting Tamil Nadu, Andhra, Kerala, and the Andaman-Nicobar Islands (2004), floods in Madhya Pradesh and Gujarat (2005), rains and floods in Maharashtra (2005), and a cyclone in Andhra Pradesh (2005).[14] These climate extremes, apart from health, also damage the public health infrastructure. India, like other developing countries, is poorly equipped to deal with weather extremes. Hence, the number of people killed, injured, or made homeless by natural disasters has been increasing rapidly.

### Health effects of more variable precipitation patterns

The Indian metropolitan city of Mumbai was besieged with India's heaviest downpour of the century in July 2005, killing nearly 600 people. According to the Indian Meteorological department, it was the heaviest ever rainfall received in a single day anywhere in India, recorded at 94.4 cm in the last 100 years. It broke the record of the previous highest rainfall at one place in India at Cherrapunjee in Meghalaya of 83.82 cm, recorded on 12 July, 1910.[11] On the other hand, Cherrapunjee in the north eastern state of Meghalaya, generally well known for being the wettest place in the world, is going through a rare rain crisis and is experiencing dry spells. This may lead to floods in some areas and drought in other areas and thus endangering food security and also affecting the quantity and quality of water. More variable rainfall patterns are likely to compromise the supply of fresh water. For example, in Kashmir (India), heat events have been increasing in the last decade. Rainfall in Srinagar appears to have been declining and Kashmir has experienced warmer than average winters, with snow melting as early as January and droughts occurring in the summer months of July and August. Water shortages have been reported during what have traditionally been wet summer months, with water having to be trucked in on occasion. There has been an increase in waterborne diseases and skin problems due to water shortages.[14] Water scarcity already affects four of every 10 people. A lack of water and poor water quality can compromise hygiene and health. This increases the risk of diarrhea, which kills approximately 1.8 million people every year, as well as trachoma (an eye infection that can lead to blindness) and other illnesses. Many diarrheal diseases vary seasonally, suggesting sensitivity to climate. In India, like in other tropics, diarrheal diseases typically peak during the rainy season. Both floods and droughts increase the risk of diarrheal diseases. Major causes of diarrhea linked to heavy rainfall and contaminated water supplies are cholera, cryptosporidium, *E. coli* infection, giardia, shigella, typhoid, and viruses such as hepatitis A.[15] In India, the threat from compromised quality of water will be larger as only 25% of the population has water piped into their dwelling, yard, or plot and one-third of the households treat their drinking water to make it potable. Half of those that treat their water strain the water through a cloth and almost one-third boil the water.[16] In 2030, the estimated risk of diarrhea will be up to 10% higher in some regions than if no climate change occurred.[12] Bundhelkhand in Uttar Pradesh had seen a reduction in rainfall in successive years from 987 mm in 2003–2004 to 240 mm in 2007–2008. Forty percent of the people have migrated from their homes and the region has faced violent conflicts over water.

### Health effects of rising sea levels

Potential effects on health due to sea level rise[17] include:

1. Death and injury due to flooding.
2. Reduced availability of fresh water due to saltwater intrusion.
3. Contamination of water supply through pollutants from submerged waste dumps.
4. Change in the distribution of disease-spreading insects.
5. Health effect on the nutrition due to a loss in agriculture land and changes in fish catch
6. Health impacts associated with population displacement.

India has a 7500 km long densely populated coast line, which is vulnerable to coastal floods, hurricanes, cyclones, and tsunami. Any increase in frequency and severity of these extreme climate events or change in coastline as projected is likely to have serious effects and can cause population displacement. These displaced people are likely to face diverse health consequences – traumatic, infectious, nutritional, psychological, and other – that occur in demoralized and displaced populations in the wake of climate-induced economic dislocation, environmental decline, and conflict situations.[14]

### Health effects of retracting glaciers

Glaciers are the source of drinking and irrigation water in the mountainous and Indo-Gangetic regions in India. Most of the states in the North, including Punjab, Haryana, Rajasthan, Uttar Pradesh, Madhya Pradesh, and the north east are dependent on river water with origin in the Himalayas. Rising temperatures may cause the snow to melt earlier and faster in the spring, shifting the timing and distribution of run-off. Projections are for a regression of the maximum spring stream-flow period in the annual cycle of about 30 days and an increase in glacier melt run-off by 33–38%.[14] These changes could affect the availability of freshwater for natural systems and human use. Excessive melt water could cause flash floods. If freshwater run-off is reduced in the summer months because of earlier melting, the result will be water insecurity and more interstate conflicts in the region.[14] Urban areas in developing countries, including India, will be more affected due to the pace of development and increasing industrialization.[18] Melting glaciers in the Himalayas may lead to glacier lake outburst floods, as occurred in Himachal Pradesh.

### Health effects due to food insecurity

Increasing temperatures and more variable rainfalls and loss of agricultural land due to flash floods are expected to reduce crop yields in many tropical developing regions, where food security is already a problem.[14,19] Simulations using dynamic crop models indicate a decrease in the yield of crops as temperature increases in different parts of India. This is likely to threaten the food security in the country where malnutrition is already an important public health problem. Malnutrition causes millions of deaths each year, from both a lack of sufficient nutrients to sustain life and a resulting vulnerability to infectious diseases such as malaria, diarrhea, and respiratory illnesses. In India, almost half of the children under age five and more than one-third of the adults are undernourished. In Bihar, Chhattisgarh, Jharkhand, Madhya Pradesh, and Orissa, more than two out of five women are undernourished. Anemia is another major nutritional health problem in India, especially among women and children. Among children between the ages of 6 and 59 months, the great majority (70%) are anemic. More than half of the women (55%) and one-fourth of the men are anemic in India. Anemia can result in maternal mortality, weakness, diminished physical and mental capacity, increased morbidity from infectious diseases, perinatal mortality, premature delivery, low birth weight, and (in children) impaired cognitive performance, motor development, and scholastic achievement.[16]

### Vector-borne diseases

Changes in climate are likely to change frequency, lengthen the transmission seasons, and alter the geographic range of important vector-borne diseases, malaria and dengue being the most important. There is historical evidence of associations between climatic conditions and vector-borne diseases. Malaria is of the great public health concerns and seems likely to be the vector-borne disease most sensitive to long-term climate change. Malaria varies seasonally in highly endemic areas. The link between malaria and extreme climatic events has long been studied in India. Early last century, the river-irrigated Punjab region experienced periodic malaria epidemics. Excessive monsoon rainfall and high humidity were identified early on as a major influence, enhancing mosquito breeding and survival. Recent analyses have shown that the malaria epidemic risk increases around five-fold in the year after an El Niño event.[20]

Mosquitoes need access to stagnant water in order to breed and the adults need humid conditions for viability. Warmer temperatures enhance vector breeding and reduce the pathogen's maturation period within the vector organism. However, very hot and dry conditions can reduce mosquito survival. Periodic epidemics of malaria occur every 5–7 seven years. Between 1994 and 1996, India experienced a sudden rise in the malaria problem, resulting in epidemics and deaths due to malaria in the states of Rajasthan, Manipur, Nagaland, and Haryana.[21] The World Bank estimates that about 577,000 disability-adjusted life years were lost due to malaria in India in 1998.[17] The malaria modeling shows that small temperature increases can greatly affect the transmission potential. Globally, temperature increases of 2–3°C would increase the number of people who, in climatic terms, are at a risk of malaria by around 3–5%, i.e. several hundred million. Further, the seasonal duration of malaria would increase in many currently endemic areas. In India, the transmission windows for malaria are predicted to increase with climate change from 4 to 6 months to 7 to 9 months in a year in Jammu and Kashmir and Madhya Pradesh and from 7 to 9 months to 10 to 12 months in Uttar Pradesh.[22]

Dengue is another important arboviral disease of humans, occurring in tropical and subtropical regions, particularly in urban settings. Since 1960, more than 50 outbreaks have been reported to or investigated by the National Institute of Communicable Diseases in India. The 1996 epidemic in New Delhi was the worst of its kind, which affected 16,517 persons and killed 545.[23] El Niño Southern Oscillation affects dengue occurrence by causing changes in household water storage practices and in surface water pooling. Between 1970 and 1995, the annual number of dengue epidemics in the South Pacific was positively correlated with La Niña conditions (i.e., warmer and wetter).[24]

Rodents, which proliferate in temperate regions following mild wet winters, act as reservoirs for various diseases. Certain rodent-borne diseases are associated with flooding, including leptospirosis, tularemia, and viral hemorrhagic diseases. Other diseases associated with rodents and ticks, and which show associations with climatic variability, include Lyme disease, tick-borne encephalitis, and Hantavirus pulmonary syndrome.[15]

### Other health effects

Increasing global temperatures affect levels and seasonal patterns of both man-made and natural air-borne particles, such as plant pollen, which can trigger asthma. About 6% of children suffer from respiratory tract infection and 2% of adults suffer from asthma.[16] Asthma deaths are expected to increase by almost 20% in the next 10 years if urgent actions to curb climate change and prepare for its consequences are not taken.[19]

Stratospheric ozone depletion is essentially a different process from climate change. However, greenhouse warming is affected by many of the chemical and physical processes involved in the depletion of stratospheric ozone. Exposure to ultra violet radiation has been implicated as a cause of skin cancer (melanoma and other types) in fair-skinned human populations living at mid to high latitudes[25,26] and also to induce immunosuppression that could influence patterns of infectious disease.[27]

## RESPONSE OPTIONS

Climate change already contributes to the global burden of disease and this contribution is expected to grow in the future. Approximately 600,000 deaths occurred worldwide as a result of weather-related natural disasters in the 1990s, some 95% of which took place in developing countries. The WHO estimated that climate change directly or indirectly contributes to about 77,000 deaths annually in Asia and the Pacific, about half of the world total attributed to climate change.[28]

The impacts of climate on human health will not be evenly distributed around the world. Developing country populations, particularly in small island states, arid and high mountain zones, and in densely populated coastal areas, are considered to be particularly vulnerable.[29] The Third Assessment Report (IPCC-2001) concluded that the extent to which human health is affected depends on the exposures of populations to climate change and its environmental consequences, the sensitivity of the population to the exposure, and the ability of affected systems and populations to adapt. Adaptation[30] can reduce sensitivity to climate change while mitigation can reduce the exposure to climate change, including its rate and extent. Both conclusions are confirmed in this assessment. Therefore, response options to protect health from effects of climate change include the mitigation as well as adaptation. Adaptation and mitigation can complement each other and together can significantly reduce the risks of climate change.[1]

The mitigation of GHGs provides a mechanism for slowing, and perhaps eventually halting, the build up of GHGs in the atmosphere. Emission of GHGs can be reduced by more efficient use of energy, reducing dependence on carbon energy and switching to low-carbon energy like solar, hydro, or wind energy, etc. Steps to reduce GHG emissions or lessen the health impacts of climate change could have positive health effects. For example, promoting the safe use of public transportation and active movement, such as biking or walking as alternatives to using private vehicles, could reduce carbon dioxide emissions and improve public health. They can not only cut traffic injuries but also air pollution and associated respiratory and cardiovascular diseases. Increased levels of physical activity can lower the overall mortality rates.[12]

A slowing of the rate of warming by mitigation of GHGs could yield important benefits in the form of reduced impacts to human health and other systems. However, the inertia in the climate system means that there will be a significant temporal lag between emission reduction and slowing in the rate of warming. Even if GHG emissions are reduced in the near future, Earth's climate will continue to change. Hence, adaptation strategies must be considered to reduce disease burdens, injuries, disabilities, and deaths. Extreme weather events can have vastly different impacts because of differences in the target population's coping capacity. For example, cyclones in Bangladesh in 1970 and 1991 are estimated to have caused 300,000 and 139,000 deaths, respectively. In contrast, Hurricane Andrew struck the United States in 1992, causing 55 deaths (although also causing around $30 billion in damages).[12] Adaptation enhances a population's coping ability and may protect against current climatic variability as well as against future climatic changes. It includes the strategies, policies, and measures undertaken now and in future to reduce the potential adverse health effects. The rebuilding and maintaining of public health infrastructure is often viewed as the “most important, cost-effective, and urgently needed” adaptation strategy.[31] This includes public health training, more effective surveillance and emergency response systems, and sustainable prevention and control programs. Education, awareness-raising, and the creation of legal frameworks, institutions, and an environment that enables people to take well-informed, long-term, sustainable decisions are also of paramount importance.

The main determinants of a community's adaptive capacity are economic wealth, technology, information and skills, infrastructure, institutions, and equity. Adaptive capacity is also a function of current population health status and pre-existing disease burdens.[12] Developed countries are better to adapt because they have the economic resources to invest and to offset the costs of adaptation. On the other hand, the developing countries who are most vulnerable (with 10% of the world's health resources, carrying 90% of the disease burden)[12] and least able to adapt to climate change are the ones least responsible for it. Equity is a key issue to the adaptation. In poor countries, the impacts of major vector-borne diseases and disasters can limit or even reverse improvements in social development.[32] Adaptive capacity is likely to be greater when access to resources within a community, nation, or the world is equitably distributed. Countries that have contributed the majority of GHG emissions should acknowledge their responsibility for generating climate change and consequent health impacts, reduce their emissions, and support under-resourced and marginal populations in adapting to climate change, to help ensure the long-term sustainability.[14]

## References

[1] (2007). Intergovernmental Panel on Climate Change. Climate Change 2007: Synthesis Report. Fourth Assessment Report.

[2] McMichael AJ (2003). Climate change and human health. Risks and responses.

[3] (2001). Intergovernmental Panel on Climate Change. Climate Change 2001. Third Assessment Report.

[4] Ravindranath NH, Sathaye J (2002). Climate Change and Developing Countries.

[5] (2004). India's Initial National Communications to the United Nations Framework Convention on Climate Change. Ministry of Environment and Forests.

[6] Kumar RK (2005). High-resolution climate change scenarios for India for the 21st century. Curr Sci.

[7] Smith JB, McCarthy JJ (2001). Vulnerability to climate change and reasons for concern: A synthesis. Climate change 2001 Impacts, adaptation and vulnerability. Contribution of Working Group II to the Third Assessment Report of the Intergovernmental Panel on Climate Change.

[8] Orissa state Disaster Mitigation Authority.

[9] World Health Organization Climate and health. Fact sheet.

[10] http://www.rediff.com/news/2003/jun/13rain.htm.

[11] Indian Meteorological Department.

[12] World Health Organisation (2003). Climate Change and Human Health: Risks and Responses. Summary.

[13] http://www.greenpeace.org/india/campaign.htm.

[14] World Health Organization (2005). Health impacts from climate variability and change in the Hindu Kush-Himalayan Region. Report of an inter-regional workshop, Mukteshwar, India.

[15] Wilson ML, Aron JL, Patz JA (2001). Ecology and infectious disease. Ecosystem change and public health: A global perspective.

[16] (2007). International Institute for Population Sciences (IIPS) and Macro International. National Family Health Survey (NFHS-3), 2005-06.

[17] Patil RR, Deepa TM (2007). Climate change: The challenges for public health preparedness and response: An Indian case study. Indian J Occup Environ Med.

[18] Lendrum DC, Corvalan C, Neira M (2007). Climate change and developing cities: Implications for environmental health and equity. J Urban Health.

[19] World Health Organisation (2008). Ten facts on climate change and health.

[20] Bouma MJ, van der Kaay HJ (1996). The El Niño Southern Oscillation and the historic malaria epidemics on the Indian subcontinent and Sri Lanka: an early warning system for future epidemics?. Trop Med Int Health.

[21] GOI Annual Report 2002-03.

[22] http://www.nplindia.org/npl/climate.

[23] Lal S, Adarsh Pankaj (2007). Text book of community medicine.

[24] Hales S, Weinstein P, Woodward A (1996). Dengue fever epidemics in the south pacific region: Driven by El nino southern oscillation?. Lancet.

[25] (1998). Environmental effects of ozone depletion: 1998 assessment.

[26] Madronich S, de Gruijl FR (1993). Skin cancer and UV radiation. Nature.

[27] Ponsonby AL, McMichael AJ, van der Mei I (2002). Ultraviolet radiation and autoimmune disease: insights from epidemiological research. Toxicology.

[28] (2002). World Health Organisation. World Health Report 2002.

[29] Woodward AJ, Hales S, Litidamu N, Phillips D, Martin J (2000). Protecting human health in a changing world: The role of social and economic development. Bull World Health Organ.

[30] Yohe G, Ebi KL, Ebi KL, Smith J, Burton I (2005). Approaching adaptation: Parallels and contrasts between the climate and health communities. Integration of public health with adaptation to climate change: Lessons learned and new directions.

[31] McMichael AJ (2003). Climate change. Comparative quantification of health risks.

[32] Bouma MJ, Kovats RS, Goubet SA, Cox JS, Haines A (1997). Global assessment of El Nino's disaster burden. Lancet.

